# Generation of an immortalized astrocytic cell line from *Abcd1*-deficient H-2K^b^tsA58 mice to facilitate the study of the role of astrocytes in X-linked adrenoleukodystrophy

**DOI:** 10.1016/j.heliyon.2021.e06228

**Published:** 2021-02-11

**Authors:** Masashi Morita, Ai Toida, Yuki Horiuchi, Shiro Watanabe, Masakiyo Sasahara, Kosuke Kawaguchi, Takanori So, Tsuneo Imanaka

**Affiliations:** a Department of Biological Chemistry, Graduate School of Medicine and Pharmaceutical Sciences, University of Toyama, Toyama, 930-0194, Japan; b Division of Nutritional Biochemistry, Institute of Natural Medicine, University of Toyama, Toyama, 930-0194, Japan; c Department of Pathology, Graduate School of Medicine and Pharmaceutical Sciences, University of Toyama, Toyama, 930-0194, Japan; d Faculty of Pharmaceutical Sciences, Hiroshima International University, Kure, Hiroshima, 737-0112, Japan

**Keywords:** Abcd1, Astrocyte, Immortalized cell, X-linked adrenoleukodystrophy, Peroxisome, Very long chain fatty acid

## Abstract

X-linked adrenoleukodystrophy (X-ALD) is an inherited metabolic disease characterized by inflammatory demyelination, and activated astrocytes as well as microglia are thought to be involved in its pathogenesis. Conditionally immortalized astrocytic cell clones were prepared from wild-type or *Abcd1*-deficient H-2K^b^tsA58 transgenic mice to study the involvement of astrocytes in the pathogenesis of X-ALD. The established astrocyte clones expressed astrocyte-specific molecules such as Vimentin, S100β, Aldh1L1 and Glast. The conditionally immortalized astrocytes proliferated vigorously and exhibited a compact cell body under a permissive condition at 33 °C in the presence of IFN-γ, whereas they became quiescent and exhibited substantial cell enlargement under a non-permissive condition at 37 °C in the absence of IFN-γ. An *Abcd1*-deficient astrocyte clone exhibited a decrease in the β-oxidation of very long chain fatty acid (VLCFA) and an increase in cellular levels of VLCFA, typical features of Abcd1-deficiency. Upon stimulation with LPS, the *Abcd1*-deficient astrocyte clone expressed higher levels of pro-inflammatory genes, such as *Il6, Nos2*, *Ccl2* and *Cxcl10*, compared to wild-type (WT) astrocytes. Furthermore, the *Abcd1*-deficient astrocytes produced higher amounts of chondroitin sulfate, a marker of reactive astrocytes. These results suggest that dysfunction of Abcd1 renders astrocytes highly responsive to innate immune stimuli. Conditionally immortalized cell clones which preserve astrocyte properties are a useful tool for analyzing the cellular and molecular pathology of ALD.

## 1 Introduction

Astrocytes are the most abundant glial cells in the central nervous system (CNS) and play important roles in maintaining normal brain homeostasis (Pfrieger and Ungerer 2011; Prat et al., 2001; Kiray et al., 2016). The supplementation of lipids such as cholesterol and polyunsaturated fatty acids by astrocytes to oligodendrocytes and neurons is essential for myelination and synaptogenesis (Camargo et al., 2017). In addition, angiogenic factors such as sonic hedgehog, vascular endothelial growth factor (VEGF), and the angiopoietins that are transferred from astrocytes to perivascular endothelial cells play an important role in the maintenance of blood brain barrier (BBB) integrity (Alvarez et al., 2011; Wang et al., 2014). Astrocytes contribute to the innate immune response, not only in infectious CNS disease but also in neurodegenerative diseases, such as Parkinson's disease, Alzheimer's disease and multiple sclerosis (MS) (Falsig et al., 2006; Liddelow et al., 2017). Therefore, the dysregulated innate immune response in astrocytes may be associated with the pathogenesis of several different neurodegenerative diseases.

X-ALD is an inherited metabolic disease that induces cerebral inflammatory demyelination (Moser et al., 2007; Kemp et al., 2012). It is caused by mutations in the *ABCD1* gene that encodes the peroxisomal ATP-binding cassette transporter ABCD1, which is involved in the transport of very long-chain fatty acids (VLCFA)-CoA into the peroxisome for β-oxidation (Morita and Imanaka 2012). Dysfunction of ABCD1 leads to increased levels of phospholipids and cholesterol esters containing VLCFAs in the CNS because of a defect in the transport of VLCFAs into peroxisomes for β-oxidation (van Roermund et al., 2008; Wiesinger et al., 2013) and/or an increase in fatty acid elongation (Morita et al., 2015; Tsuji et al., 1984). In the mouse brain, Abcd1 is highly expressed in astrocytes and microglia, moderately in oligodendrocytes, and scarcely in neurons (Troffer-Charlier et al., 1998). In X-ALD patients, activated astrocytes and microglia are located throughout the white matter before demyelination and inflammation occur (Eichler et al., 2008; Gortz et al., 2018). Therefore, it has been speculated that astrocytes and microglia with dysfunctional ABCD1 could play a role as initiators of inflammatory demyelination. Although the mechanism by which the accumulated VLCFA causes inflammatory demyelination is still obscure (Berger et al., 2014; Wiesinger et al., 2015), the accumulation of VLCFA may contribute to mitochondrial damage and the generation of reactive oxygen species (ROS), which in turn leads to the production of proinflammatory cytokines (Powers et al., 2005; Gilg et al., 2000). Gortz et al. (2018) have suggested that certain pathogenic factors that trigger cellular stress in astrocytes may be involved in the activation of microglia and initiation of inflammatory demyelination in X-ALD. However, the specific contribution of astrocytes to the pathogenesis of X-ALD is still largely undetermined.

Primary astrocytes from neonatal mouse brain are normally used to analyze the function of astrocytes (Lange et al., 2012). However, even highly purified primary astrocytes still contain a few microglia, which may interfere with the investigation of the inflammatory responses mediated by astrocytes (Saura 2007) (Hamby et al., 2006). We have purified astrocytes from mixed glial cells by repeated passages and treatment with inhibitors such as cytosine arabinoside (Ara-C) and L-leucine methyl ester, and most of the microglia are removed by this protocol (Pont-Lezica et al., 2013). However, the possibility that a small number of residual microglia remain exert an influence on the inflammatory reactions in astrocytes cannot be ruled out. To exclude the possibility, we generated conditionally immortalized astrocytic cell clones from wild-type or *Abcd1*-deficient H-2K^b^tsA58 transgenic mice that carry the temperature sensitive SV40 large T antigen under the control of the interferon-γ (IFN-γ) H-2Kb promoter (Jat et al., 1991; David-Watine et al., 1990). Under a permissive condition of 33 °C in the presence of IFN-γ, purified astrocytes expressing SV40 large T antigen proliferated vigorously. In this study, we prepared conditionally immortal astrocyte clones and conducted a comparative analysis between wild-type and *Abcd1*-deficient immortalized astrocytes.

## 2 Materials and methods

### 2.1 Materials

[1-^14^C]lignoceric acid (53 mCi/mmol) was purchased from Moravek Biochemicals (Brea, CA). Western Lightning Plus-ECL, a Western blotting detection system was purchased from PerkinElmer (Waltham, MA). The rabbit anti-ABCD3 antibody that was raised against the COOH-terminal 15 amino acids of rat Abcd3 (Imanaka et al., 1996) and the rabbit anti-ABCD1 antibody that was raised against the COOH terminal 24 amino acids of human ABCD1 (Morita et al., 2012) were used in this study. Other antibodies were purchased from commercial sources, including a polyclonal rabbit anti-Abcd2 antibody (ABclonal, Inc., Tokyo, Japan), a polyclonal rabbit anti-catalase antibody (Rockland Immunochemicals, Inc, Limerick, PA), polyclonal rabbit anti-S100β antibody (GeneTex, Inc., Alton Pkwy Irvine, CA), mouse monoclonal anti-SV40LT antibody (Thermo Fisher Scientific, Waltham, MA, Clone PAb101), mouse monoclonal anti-A2B5 antibody (Miltenyi Biotec GmbH, Gladbach, Germany, Clone 105-HB29), mouse monoclonal anti-chondroitin sulfate antibody (Sigma Aldrich Co. LLC, Saint Louis, MO, Clone CS-56), polyclonal rabbit anti-glial fibrillar acidic protein (GFAP) antibody (Agilent Technologies, Santa Clara, CA), rabbit anti-Iba1 antibody (Wako Chemicals Ltd. Osaka, Japan), mouse monoclonal anti-nestin antibody (Thermo Fisher Scientific, Waltham, MA, Clone Rat-401), and mouse anti-vimentin antibody (Abcam, Cambridge, CB2 0AX, UK, Clone RV202). The secondary antibodies including Alxa555-labeled goat anti-rabbit and anti-mouse IgG, Alxea488-labeled goat anti-rabbit and anti-mouse IgG, were from Thermo Fisher Scientific.

### 2.2 Mice

The *Abcd1*-deficient mouse was generated at Kyushu University by Kobayashi et al. (1997). *Abcd1*-deficient mice backcrossed to C57BL/6J for 10 generations were kindly provided by Dr. Hashimoto of the National Institute for Longevity Sciences, Aichi, Japan. Wild-type mice (C57BL/6J) were purchased from Japan SLC Inc. and H-2k^b^tsA58 transgenic mice were obtained from Charles River Laboratories (Shizuoka, Japan). To generate *Abcd1*-deficient H-2k^b^tsA58 transgenic mouse, an H-2k^b^tsA58 transgenic female mouse was crossed with an *Abcd1*-deficient male mouse lacking the *Abcd1* gene on the X-chromosome. Mice were maintained at the animal facility of the University of Toyama under controlled conditions with food and water provided *ad libitum*. All of the procedures used in the animal laboratory research were approved by the University Committee for Animal Use and Care at the University of Toyama.

### 2.3 Primary culture of mouse astrocytes

Mixed glial cultures were prepared from neonatal mouse brain according to the method of Sawada et al. (1992) with some modifications. Briefly, the cerebrum from 1- to 2-day-old postnatal mice was transferred to ice-cold HBSS/EMEM with 10% fetal calf serum (FCS; Biowest, France) (1:1, v/v) and the meninges were carefully removed. The cerebrum was minced and digested with papain (0.15 units/mL) at 37 °C for 30 min. After incubation, the cells were pelleted by centrifugation, suspended with repeated pipetting and passed through a metallic mesh (300 μm). The dispersed cells were seeded on a 25T culture plate and cultured at 37 °C in humidified 5% CO_2_. Medium was replaced every 3 days and confluency was achieved after 10–12 days.

Mixed glial cells cultures were vigorously shaken for a few minutes with a replaced serum-free EMEM medium to remove adherent oligodendrocytes and microglia from the astrocytic monolayers. The residual astrocytic cells were removed by mild trypsinizaion according to the method of Saura et al. (2003) as follows. The residual astrocytic cells were washed 3 times with serum-free EMEM medium and treated with a mixture of 0.25% trypsin/EDTA solution and serum-free EMEM medium (1:1, v/v) at 37 °C for up to 60 min. The sheet-like astrocytes detached from the culture dish were collected by centrifugation and resuspended in 0.25% trypsin/EDTA solution for few minutes to bring them to a single cell population. The cells were resuspended in EMEM/10%FCS and transferred to non-coated bacterial culture dishes. After incubation at 37 °C for more than 2 h, the astrocytes were detached from the dish, leaving firmly attached microglia, and were transferred to a culture dish and cultured for an additional 4 or 5 days. Under confluent conditions, medium was replaced daily with a medium containing Ara-C (final conc. 8 μM) for 5 days to remove the microglia. In addition, astrocyte culture was treated with LME (final conc. 50 mM) for 1 h to kill the microglia (Pont-Lezica et al., 2013). After being washed 3 times with HBSS to remove the LME completely, they were incubated with EMEM/10%FCS for more than 24 h before the experiment. The purified astrocytes were used as primary astrocytes.

### 2.4 Preparation of immortalized astrocytes and treatment with lipopolysaccharide

Immortalized astrocytes were prepared from mixed glial cells taken from H-2k^b^tsA58 transgenic mice. A mixed glial culture derived from a male mouse was determined by genotyping. A mixed glial culture expressing both the *H-2kb* and *Sry* genes was used for further experiments (Suppl. Fig. 1). After removing microglia from the mixed glia as described above, primary astrocytes were incubated under conditions in which the medium contained 25 units/ml of mouse IFN-γ (Miltenyi Biotec Inc. CA, USA) and the culture temperature was shifted to 33 °C (i.e. the permissive condition). Immortalized astrocytes were cloned by colony formation under the permissive condition. The cloned immortalized cell lines were maintained and expanded by culturing, again under the permissive condition. When the immortalized astrocytes were introduced to differentiation in a non-growth state, they were incubated with EMEM/10% FCS medium in the absence of IFN-γ at 37 °C for more than 7 days (i.e. the non-permissive condition).

Before the immortalized cells were treated with lipopolysaccharide (LPS from 0111:B4 γ-irradiated *Escherichia coli*) at 1 μg/ml, they were rinsed with serum-free medium 3 times, then the medium was replaced with EMEM medium containing 1% FCS and incubated for 12 h in the same medium. Under the permissive condition, IFN-γ was added to the medium to augment cell proliferation by enhancing the expression of the H-2Kb class I promoter. As IFN-γ is known to stimulate the inflammatory reaction in astrocytes, immortalized astrocytes were incubated under the non-permissive condition for more than 1 week before the experiment.

### 2.5 Immunofluorescence

Immunofluorescence analysis was performed as described previously (Morita et al., 2013). Cells were seeded onto glass coverslips and fixed with 4% formaldehyde in PBS for 20 min at room temperature. In some experiments, cells were fixed with cold methanol for 5 min followed by cold acetone for 1 min. The antibodies against SV40LT (1:200), vimentin (1:250), GFAP (1:200), S100β (1:250) and nestin (1:100) were used as the primary antibodies. Alxa488-conjugated goat anti-rabbit (1:500) and Alex555-conjugated goat anti-mouse (1:500) were used as the secondary antibodies. All of the antibodies were diluted with PBS containing 0.02% bovine serum albumin. The cells were mounted in Vectarshield with DAPI (Vector Laboratories, Burlingame, CA) and examined under fluorescence microscopy (Olympus, AX80TRF-65, Tokyo, Japan) or LSM780 confocal microscopy (Carl Zeiss Microscopy, Jena, Germany).

Staining with antibodies against A2B5 or chondroitin sulfate was carried out at 4 °C for 30 min on live cultures before fixation (Redies et al., 1991). The cells on the coverslips were rinsed with serum-free EMEM medium and incubated for 24 h in the same medium. The cells were incubated with anti-chondroitin sulfate antibody (1:200) or anti-A2B5 antibody (1:5) for 30 min at 4 °C. The cells were washed 3 times with serum-free EMEM medium followed by incubation with Alexa555-conjugated anti-mouse Ig antibody. The cells were fixed with cold acetic acid/ethanol (5:95, v/v) for 5 min at -20 °C and mounted as described above.

### 2.6 Quantitative real-time PCR

Total RNA from cultured cells was isolated using Isogen II (Nippon Gene Co., Ltd., Tokyo, Japan) according to the manufacturer's protocol. First strand cDNA was synthesized using random hexamer primers and a cDNA synthesis kit (Toyobo Co., Ltd. Osaka, Japan). Quantitative PCR and melting curve analysis were performed with a Brilliant SYBR Green QRT-PCR Kit (Toyobo Co., Ltd. Osaka, Japan) using a Mx3000P QPCR system (Agilent Technologies, Santa Clara, CA). The PCR conditions included an initial denaturation at 95 °C for 60 s, followed by 40 cycles of 95 °C for 15 s and 60 °C for 60 s. The relative quantitative expression of genes was calculated by the comparative Ct method, and normalized against *Ppib* mRNA. Results are presented as the mean value ±SD. The results for each gene are expressed in relation to the control, which was arbitrarily taken to be equal to 1. The primers used in this study are listed in Table 1.

**Table 1:** Primer sequences for PCR analysis.

Gene	Forward (5′ to 3′)	Reverse (5′ to 3′)
*Gfap*	ACCGCATCACCATTCCTGTAC	TGGCCTTCTGACACGGATTT
*Glast*	GATGCTGCAGATGCTGGTCTT	TATCTAGGGCCGCCATTCCT
*Ald1l1*	AAGTCACCCCTTATCATCTTTGCT	CCCTACTTCCTCCACCACTTTCT
*S100b*	AGGGTGACAAGCACAAGCTGA	GTCCACCACTTCCTGCTCCTT
*Vim*	GCTGCAGGCCCAGATTCAG	GCAGTGAGGTCAGGCTTGGA
*Ccl2*	TCTGTGCTGACCCCAAGAAG	GGTTGTGGAAAAGGTAGTGGATG
*Cxcl10*	AATCATCCCTGCGAGCCTATC	GCTCTCTGCTGTCCATCCATC
*Il1b*	CAACAGTGGTCAGGACATAATTGAC	GGCAAGGAGGAAAACACAGG
*Il6*	CTGGGAAATCGTGGAAATGAG	GGACTCTGGCTTTGTCTTTCTTG
*Tnfa*	CTCTGTGAAGGGAATGGGTGT	TGTCCCAGCATCTTGTTTCT
*Nos2*	GCAGCTACTGGGTCAAAGACAA	TCTCTGCCTATCCGTCTCGTC
*Sry*	CCATGTCAAGCGCCCCATGA	GTAAGGCTTTTCCACCTGCA-
*H-2Kb*	AGCGCTTGTGTCGCCATTGTATTC	GTCACACCACAGAAGTAAGGTTCC
*18sRNA*	CGTTCTTAGTTGGTGGAGC	TAAGGGCATCACAGACCT
*Ppib*	TGGAGAGCACCAAGACAGACA	TGCCGGAGTCGACAATGAT

### 2.7 Enzyme-linked immunosorbent assay

IL-6 and IL-1β in culture supernatants was assessed by a sandwich enzyme-linked immunosorbent assay. In brief, 96-well enzyme immunoassay plates were incubated overnight with purified anti-mouse-IL-6 antibody (BioLegend 504501) or purified anti-mouse/rat-IL-1β antibody (BioLegend 503501) as capture antibody. After washing with PBS containing 0.05% Tween-20 and blocking with 1% BSA in PBS for 1 h, plates were incubated with samples and standards for 2 h. Next, biotin-anti-mouse IL-6 antibody (BioLegend 504601) or biotin-anti-mouse IL-1β antibody (BioLegend 515801) was added and incubated for 1 h. Bound IL-6 or IL-1β was detected using Streptavidin-HRP development (BioLegend 405210) and absorbance at 450 nm was read using FilterMaxF5 (Molecular Devices, CA, US). The assay was sensitive to 8 pg/mL IL-6 and 30 pg/mL IL-1β.

### 2.8 Genotyping

The genotype of the cultured cells was determined by PCR using a MightyAmp Genotyping Kit (Takara, Shiga, Japan) according to the manufacturer's protocol. Mous*e H-*2K^b^ and *Sry* genes were amplified using the sense and antisense primers listed in Table 1. Amplified products were electrophoresed in 2% agarose gels and stained with ethidium bromide to detect the amplicons. Primary astrocytes expressing both genes were used for cloning.

### 2.9 Other methods and statistical analysis

Analysis of the fatty acid content was performed by gas-liquid chromatography as described previously (Morita et al., 2005). Fatty acid β-oxidation was measured as described previously (Morita et al., 2012). Immunoblotting was performed using the ECL Plus Western blotting detection reagent (Morita et al., 2012). The protein concentration was determined by the Lowry method using bovine serum albumin as the standard (Lowry et al., 1951). *P* values were calculated using unpaired student's *t*-test. A *p-*value of <0.05 was used as the criterion for statistical significance.

## 3 Results

### 3.1 Establishment of astrocytic clones

**Figure 1:**
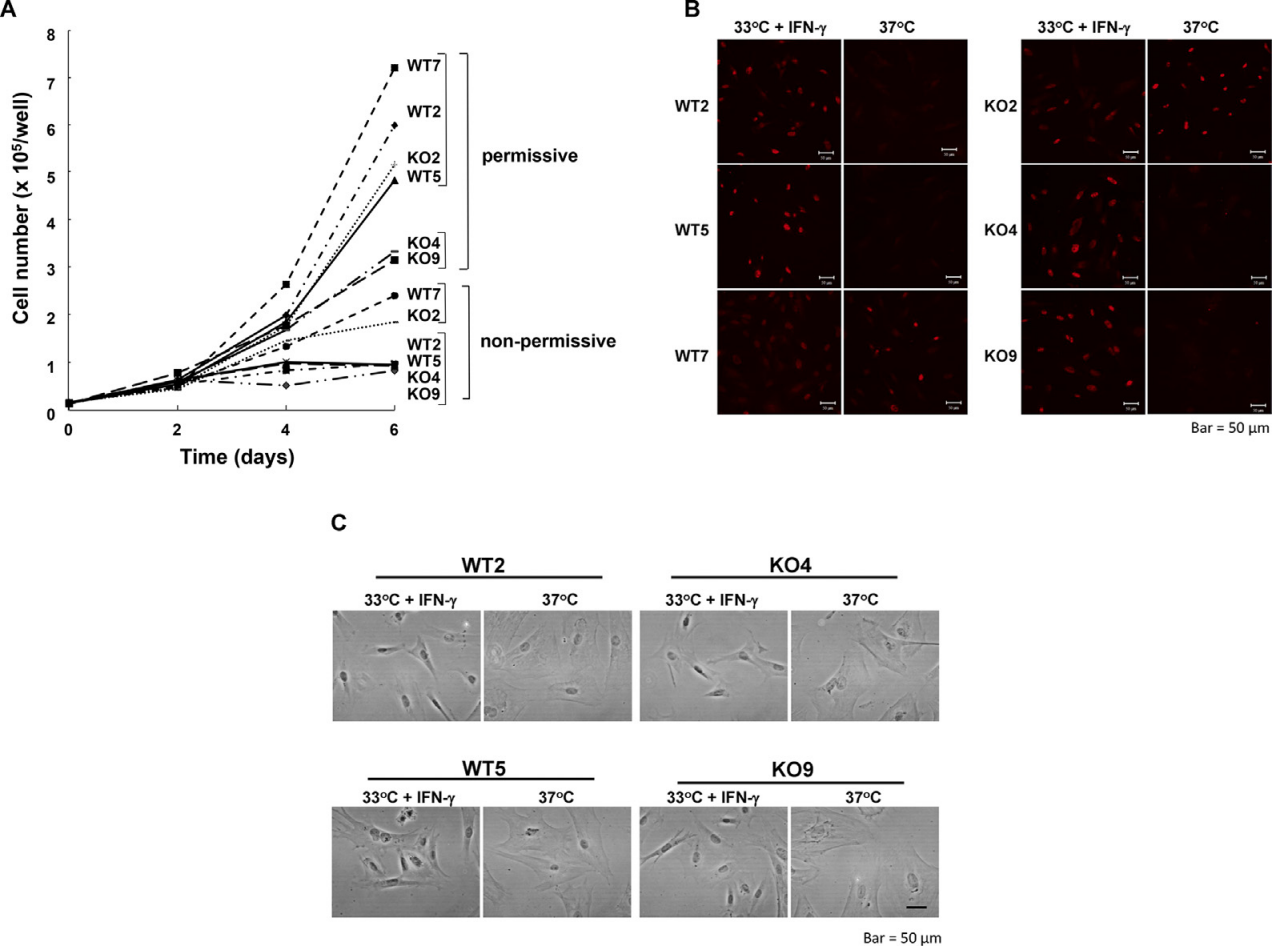
Characteristics of immortalized astrocytic cell clones. A) Growth of each cell clone under a permissive or non-permissive condition. Each of three immortalized cell clones, wild-type (WT1, WT2, WT3) and *Abcd1*-deficient (KO1, KO2, KO3), which were prepared form H-2k^b^tsA58 transgenic mice brain, were cultured under a permissive (at 33 °C in the presence of interferon-γ) or non-permissive condition (at 37 °C in the absence of interferon-γ). Astrocytes were plated at 2.5 x 10^4^ cells per well in 6-well culture plates and grown for 6 days. The total cell number in each well was determined using a hematocytometer every 2 days. B) SV40LT expression in immortalized clones under a permissive or non-permissive condition. The immortalized clones (WT2, WT5, WT7, KO2, KO4 and KO9) were cultured under a permissive or non-permissive condition for 7 days. After fixation, cells were incubated with an anti-SV40LT primary antibody followed by an anti-rabbit Ig-Alexa555 secondary antibody and observed under fluorescence microscopy. Bar, 50 μm. C) Phase-contrast micrographs of immortalized clones. The immortalized clones (WT2, WT5, KO4 and KO9) were cultured under a permissive condition or non-permissive condition for 7 days. The cells were observed under phase-contrast light microscopy. Bar, 50 μm.

Female *Abcd1*-deficient (homozygous) mice were mated with male H-2k^b^tsA58 transgenic mice to generate male *Abcd1*-deficient (hemizygous) H-2k^b^tsA58 transgenic mice. Mixed glial cultures expressing both the *H-*2K^b^ and *Sry* genes (Suppl. Fig. 1) were used to prepare immortalized astrocytic cell clones. In this study, 3 immortalized astrocytic clones were prepared from the wild type (designated as WT2, WT5 and WT7) and *Abcd1*-deficient (KO2, KO4 and KO9) H-2k^b^tsA58 transgenic mouse brain. These clones grew exponentially under the permissive conditions (i.e. at 33 °C in the presence of IFN-γ), although the growth of KO4 and KO9 was relatively slow (Figure 1A). Under the non-permissive condition at 37 °C without IFN-γ, the growth of WT2, WT5, KO4 and KO9 was completely halted, whereas that of WT7 and KO2 was attenuated. SV40LT disappeared from WT2, WT5, KO4 and KO9 under the non-permissive condition, while WT7 and KO2 still expressed the SV40LT-antigen (Figure 1B). Under the non-permissive condition, these clones in each of two cell lines (WT2, WT5 and KO4, KO9) became enlarged upon growth cessation and appeared stellate in shape, resembling primary astrocytes, in sub-confluent cultures (Figure 1C). The SV40LT-antigen in these cell lines decreased in a time-dependent manner under the non-permissive condition with growth cessation, indicating that the temperature-sensitive growth characteristic is due to the activity of the SV40LT-antigen. In the following experiments, WT5 and KO9 were used for analysis.

### 3.2 Characterization of the astrocytic clones

**Figure 2:**
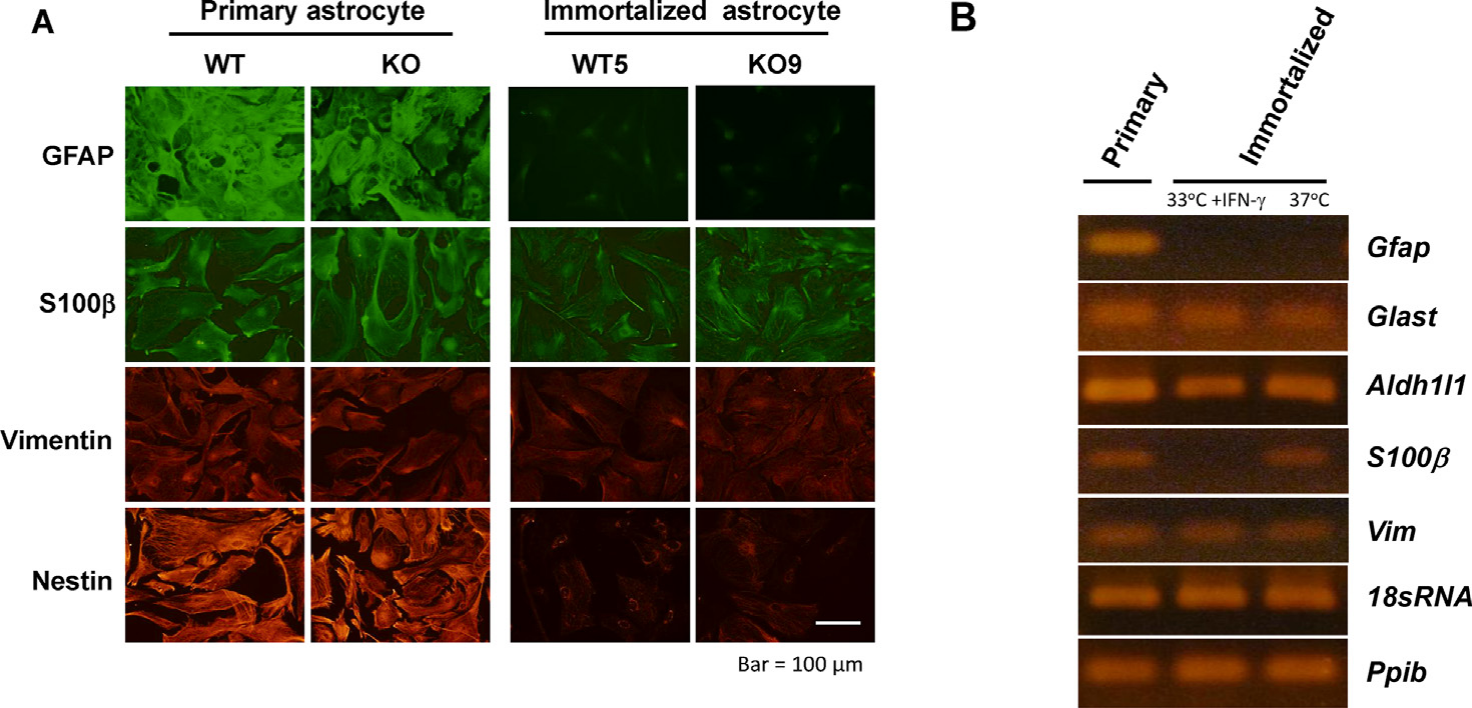
Expression of astrocytic markers in the immortalized clones. Primary astrocytes (WT and KO) and immortalized astrocyte clones (WT5 and KO9) cultured under non-permissive condition for 7 days were analyzed by immunofluorescence (A). Cells were stained with anti-GFAP, anti-S100β, anti-vimentin or anti-nestin primary antibodies followed by staining with anti-mouse-Ig-Alexa555 or anti-rabbit-Ig-Alexa488 secondary antibodies. They were observed under fluorescent microscopy. Bar, 50 μm. The gene expression of astrocytic markers in wild-type primary and immortalized astrocytes (WT5) cultured under a permissive or non-permissive condition was determined by PCR (B). Total RNA prepared from primary astrocytes and immortalized astrocytes under a permissive or non-permissive condition was reverse-transcribed to cDNA. PCR was performed using cDNA as the template with the primer pairs listed in Table 1. Amplified products were electrophoresed in 2% agarose gels and stained with ethidium bromide to detect the amplicons.

The expression levels of the astrocyte marker proteins in WT5 and KO9 under the non-permissive condition were evaluated by immunofluorescence analysis. The level of the major astrocytic marker GFAP in these clones was far less than that seen in primary astrocytes (Figure 2A). In contrast, the marker of immature astrocytes nestin was detected in these clones, although the expression was lower than that in primary astrocytes. S100β and vimentin, a cytosolic Ca^2+^ binding protein typically used as a glial marker and a classical intermediate filament component in immature astrocyte, respectively, were expressed at a level similar to that in primary astrocytes. In immortalized astrocytic cell clone, the expression of astrocyte-specific genes such as *Gfap* was hardly detected under either the permissive or non-permissive condition, whereas *S100β* was detected under the non-permissive but not the permissive condition (Figure 2B). *Glast* and *Aldh1l1*, a glutamate/aspartate transporter and aldehyde dehydrogenase family 1 member L1, respectively, are well-described astrocyte markers (Cahoy et al., 2008). *Glast* and *Aldh1l1,* as well as *S100β* and *Vim*, were expressed under both the permissive and non-permissive conditions, confirming that the immortalized astrocytic cell clone possess the characteristic of astrocytes. Furthermore, the astrocyte clones were immunohistochemically negative for A2B5, the marker of oligodendrocyte-type 2 astrocyte progenitor cells (data not shown). Thus, the immortalized astrocyte cell clones, which were GFAP- and A2B5-negative but nestin-, vimentin-, and S100β-positive, were found to have a phenotype resembling immature or reactive type I astrocytes.

### 3.3 VLCFA metabolism in astrocytic clones

**Figure 3:**
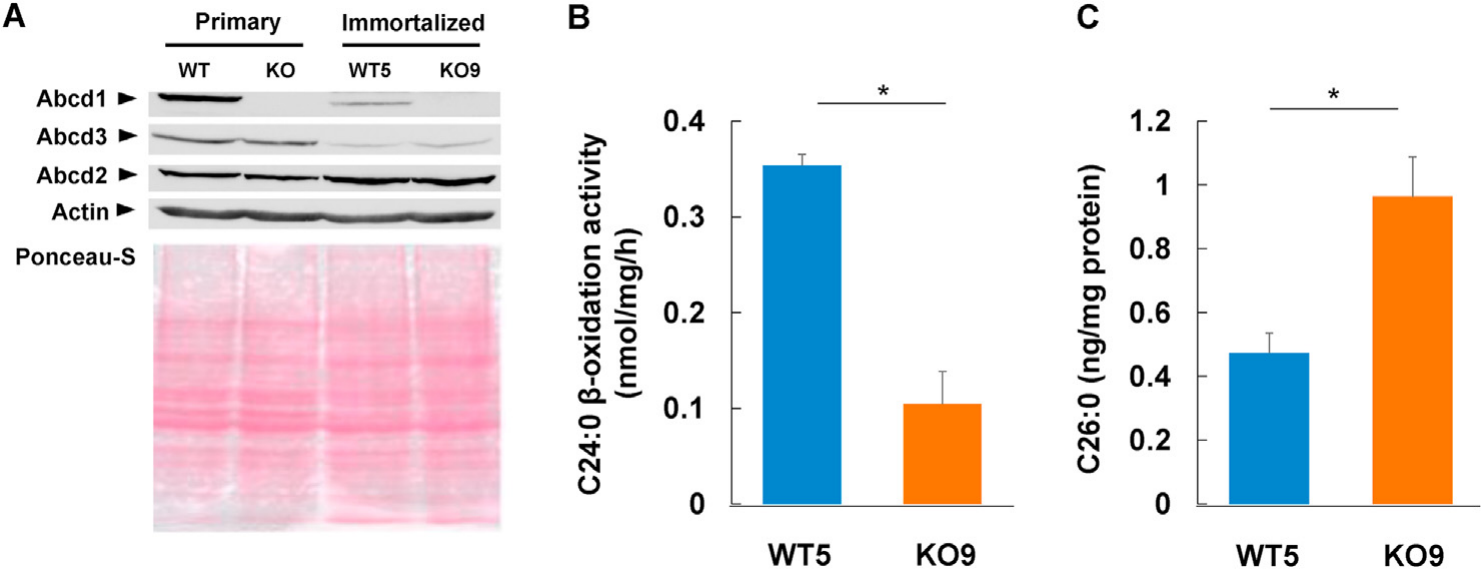
VLCFA β-oxidation activities and cellular VLCFA levels in the immortalized astrocytes. The total cellular proteins (150 μg protein per lane) that were prepared from the primary astrocytes (WT and KO) as well as the immortalized astrocytes (WT5 and KO9) were analyzed by immunoblotting using an anti-Abcd1, anti-Abcd2, anti-Abcd3 and anti-actin primary antibody, respectively (A). The transferred proteins were checked by staining with Ponceau S. Full images were represented in supplementary materials (Suppl. Fig. 2). VLCFA β-oxidation activities in the immortalized astrocytes (WT5 or KO9) were measured using [1-^14^C]C24:0 as the substrate (B). The VLCFA β-oxidation activities of WT5 (gray bars) and KO9 (black bar) are expressed as nmol/mg/h. The results are presented as the means ± S.D.; n = 4. (∗, *p* < 0.02 vs. the WT5). Total fatty acids were extracted from immortalized astrocytes (WT5 or KO9) and analyzed by gas-liquid chromatography (C). The values of WT5 (gray bars) and KO9 (black bar) are expressed as C26:0 ng/mg protein. The data are the mean **±** S.D. (∗, *p* < 0.02 vs. the WT).

We next analyzed the effect of Abcd1-deficiency in immortalized cell clones. In mammals, the three peroxisomal ABC proteins Abcd1, Abcd2 and Abcd3 reside in the peroxisomal membrane. Abcd1 and its closest homolog, Abcd2, are mainly involved in the metabolic transport of saturated and unsaturated VLCFA-CoA into peroxisomes, respectively. In contrast, Abcd3 transports long chain fatty acids, bile acid intermediates and branched chain fatty acids (Morita and Imanaka 2012). As Abcd1 transports VLCFA-CoA into peroxisomes, Abcd1 dysfunction results in a decrease in peroxisomal VLCFA β-oxidation activity and an increase in VLCFA level. In immortalized astrocytic cell clone (WT5), the level of Abcd1 and Abcd3 proteins was relatively low in comparison with wild-type primary astrocytes (Figure 3A). In contrast, the level of Abcd2 protein in immortalized astrocytes was higher than that in primary astrocytes.

The C24:0 β-oxidation activity per cellular protein in WT5 (0.35 ± 0.01 nmol/mg/h) was relatively low compared with that in wild-type primary astrocytes (1.18 ± 0.12 nmol/mg/h) and the C26:0 level per cellular protein in WT5 (0.47 ± 0.06 ng/mg protein) was higher than that in wild-type primary astrocytes (0.12 ± 0.03 ng/mg protein). However, KO9 showed a significant decrease in C24:0 β-oxidation activity and an increase in the cellular level of C26:0 compared with WT5 (Figure 3B and C), indicating that the immortalized astrocytes show typical metabolic abnormalities caused by the lack of Abcd1 protein.

### 3.4 Inflammatory state of the astrocytic clones

**Figure 4:**
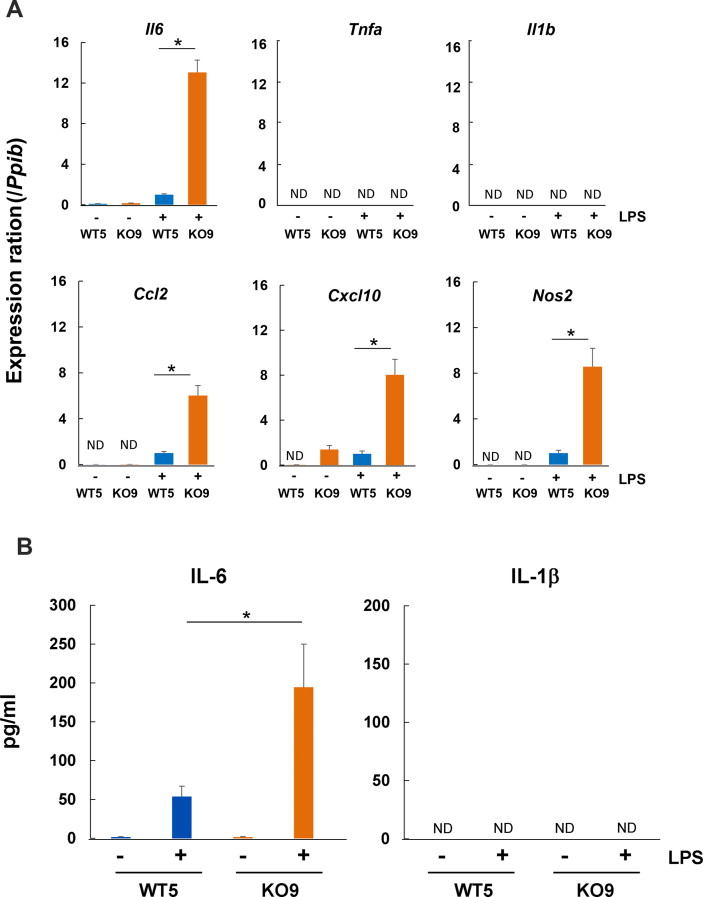
Proinflammatory gene expression in immortalized astrocytes upon LPS stimulation. A) The immortalized cells (WT5 or KO9) under the non-permissive condition were cultured with EMEM/1% FCS for 24 h and treated with 1 μg/ml of LPS for 12 h. The gene expression of *Il6, Tnfa, Il1β, Ccl2, Cxcl10* and *Nos2* was analyzed by real-time PCR. Values are normalized to the expression of *cyclophilin A*. The expression level of WT5 after LPS stimulation in the respective genes was adjusted to 1. n = 5 for all groups. (∗, *p* < 0.05). ND, not detected. B) The level of IL-6 and IL-1β in culture medium was analyzed by ELISA. The immortalized cells were incubated as in A. n = 5 for all groups. (∗, *p* < 0.05). ND, not detected.

We next analyzed the inflammatory state of the *Abcd1*-deficient immortalized astrocytes (KO9) in comparison with wild-type immortalized astrocytes (WT5) under the non-permissive condition. The expression of inflammatory-related genes such as *Il6, Tnfa, Il1b, Ccl2, Cxcl10* and *Nos2* was very low or not detected in the immortalized cells before stimulation with LPS. Upon LPS stimulation, these pro-inflammatory genes such as *Il6, Ccl2, Cxcl10* and *Nos2* were strongly upregulated, and the expression levels were markedly higher in KO9 (Figure 4A). In contrast, the expression of *Tnfa* and *Il1b* genes was insensitive to LPS stimulation. Figure 4B showed that the secretion of IL-6 was detected in the culture medium only when they were treated with LPS, and the level in IMT-KO9 was significantly higher than that in IMT-WT5. In contrast, the secretion of IL-1β was undetectable in either IMT-WT5 or IMT-KO9 even in the presence of LPS.

**Figure 5:**
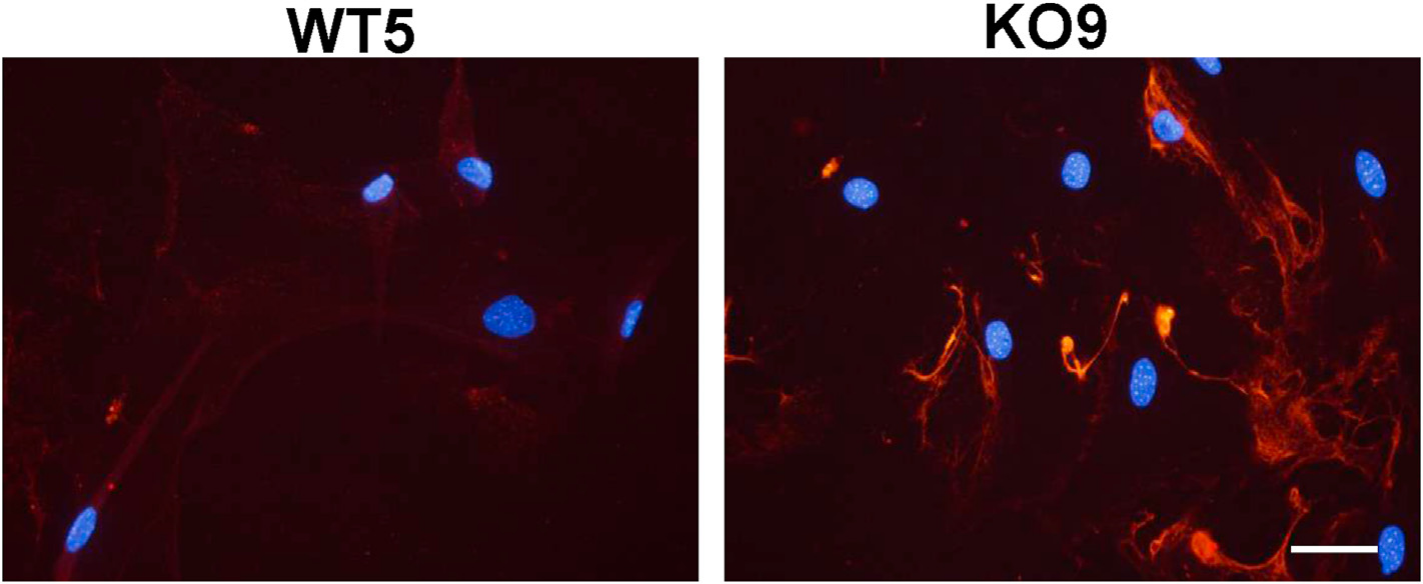
Expression of chondroitin sulfate in immortalized astrocytes. The immortalized cells (WT5 or KO9) grown on coverslips were reacted with an anti-CS56 primary antibody for 1 h and fixed. The fixed cells were stained by an Alexa555-labeled secondary antibody and observed under fluorescent microscopy. Nucleuses were stained with DAPI. Bar, 50 μm.

In addition, the extracellular region in KO9 was highly stained with anti-chondroitin sulfate monoclonal antibody (Figure 5), suggesting that KO9 express larger amounts of chondroitin sulfate proteoglycan (CSPG) than WT5. The extracellular CSPG was detected in immortalized cells but not in primary astrocytes (data not shown). As extracellular CSPG is known to be a marker of reactive astrocytes, KO9 may be in a more reactive state than WT5.

Taken together, this study demonstrates that *Abcd1*-deficient astrocytes are poised to respond to innate immune stimuli through the production of proinflammatory mediators, suggesting a possible linkage between the dysregulated astrocyte activity and the pathogenesis of X-ALD.

## 4 Discussion

The objective of this study was to establish *Abcd1*-deficient astrocytes for the purpose of studying the involvement of astrocytes in the pathogenesis of X-ALD. Recently, astrocytes have come to be recognized as an important regulator of inflammatory immune responses (Liddelow and Barres 2017; Rothhammer and Quintana 2015). In neurodegenerative diseases, reactive astrocytes produce an array of inflammatory cytokines and chemokines which open the BBB, leading to the infiltration of peripheral immune cells into the brain (Choi et al., 2014). In addition, activated astrocytes function as antigen-presenting cells activate T cells, resulting in T cell-mediated neuroinflammation (Gimsa et al., 2013). In cerebral X-ALD, reactive astrocytes as well as reactive microglia are distributed throughout the white matter prior to macrophage/lymphocyte infiltration (Gortz et al., 2018). Thus, it is tempting to speculate that the up-regulation of inflammatory mediators in astrocytes may promote the development of X-ALD (Eichler et al., 2008; Hein et al., 2008; Schluter et al., 2012). However, how astrocytes enter into a more inflammatory active state in the brain of X-ALD has yet to be elucidated.

The use of ts58 mutant SV40 large T antigen in conditional immortalization has been described by Groves et al. (1993) and others (Noble et al., 1995; Langley et al., 2009). The conditionally immortalized astrocyte cells offer several advantages. They enable the supply of a number of purified astrocytic cells, reduce the time and labor required for preparing astrocytes in each experiment, regulate differentiation by the culture temperature and also exclude the involvement of other cell types in the primary astrocyte culture. In this study, conditionally immortalized astrocytic clones were established that can grow for extended periods under the permissive condition, but undergo growth inhibition and morphological change under the non-permissive condition (Figure 1). The immortalized astrocytic clones in the present study scarcely expressed GFAP, but expressed other astrocyte-specific proteins (such as S100β) and genes (such as *Glast* and *Aldh1l1*) (Figure 2A and B). It has been reported that immortalized astrocytes express lower levels of GFAP compared to non-immortalized astrocytes (Frisa et al., 1994). In addition, the conditionally immortalized astrocytes prepared from H-2K^b^tsA58 transgenic mice displayed several biological and biochemical properties associated with glial scars (Groves et al., 1993; Noble et al., 1995; Langley et al., 2009). Similar to previous studies (Ahlemeyer et al., 2013; Seidman et al., 1997; Miller et al., 1985), the immortalized astrocytes in the present study did not express a maker for oligodendrocyte-type 2 astrocyte progenitor cells, but expressed vimentin and nestin rather than GFAP, indicating that the established clones are phenotypically similar to reactive type I astrocytes.

The expression level of the peroxisomal membrane proteins (Abcd1, Abcd2 and Abcd3) was considerably different between immortalized astrocytes and primary astrocyte (Figure 3A). Interestingly, the level of Abcd1 and Abcd3 in immortalized astrocytes was lower than that in primary astrocytes, whereas Abcd2 protein was rather high in immortalized astrocytes. This result suggests that dysfunction of Abcd1 in immortalized astrocytes may be partly complemented by Abcd2. *Abcd1*-deficient immortalized astrocytes displayed a clearly evident decrease in VLCFA β-oxidation and increase in the VLCFA level (Figure 3B and C), which allowed characterization of the inflammatory state in these cells.

It is known that under inflammatory conditions, astrocytes secrete soluble mediators, such as CXCL10, CCL2 and IL-6, that may be involved in the disease mechanisms underlying inflammatory demyelination (Farina et al., 2007). CCL2 maintains BBB integrity by stabilizing endothelial tight junctions, and when disturbed, ultimately enables leukocyte penetration into the brain parenchyma. CXCL10, mainly released by activated astrocytes, is also implicated in the recruitment of leukocytes into the CNS (Fife et al., 2001). In the present study, the gene expression of *Il6*, *Ccl2, Cxcl10* and *Nos2* in *Abcd1*-deficient immortalized astrocytes (KO9) was greatly enhanced by LPS stimulation as compared to wild-type immortalized astrocytes (WT5) (Figure 4A) and the secretion of IL-6 was higher in KO9 than WT5 (Figure 4B), indicating that Abcd1-deficiency keeps astrocytes in a primed state. In chronic neurodegeneration, astrocytes are suggested to be primed in such a manner as to produce exaggerated amounts of chemokines in response to pro-inflammatory cytokines such as IL-1β and TNF-α (Hennessy et al., 2015). Similar to chronic neurodegenerative disease, the response to cytokines and LPS was shown to be higher in iPSC (induced pluripotent stem cell)-derived astrocytes from cerebral ALD fibroblasts (Baarine et al., 2015). It is thus assumed that *Abcd1*-deficiency predisposes astrocytes to a pro-inflammatory state, where additional stimuli result in an exaggerated pro-inflammatory response. In addition, it has been reported that in cerebral ALD the induction of proinflammatory cytokines is paralleled by the up-regulation of the *NOS2* gene that encodes for inducible nitric oxide synthase (Gilg et al., 2000; Paintlia et al., 2003). It has been reported that lipid-peroxidation level in *Abcd1*-deficient astrocytes was significantly higher than that in wild-type astrocytes (Kruska et al., 2015), suggesting that *Abcd1*-deifient astrocytes are more sensitive to oxidative stress. In this study, *Nos2* gene expression in *Abcd1*-deficient immortalized astrocytes (KO9) was also enhanced by LPS stimulation (Figure 4), suggesting that oxidative stress may be involved in the augmented inflammatory response.

In addition to the higher inflammatory response, we found that *Abcd1*-deficient immortalized astrocytes (KO9) produced larger amounts of CSPG compared to wild-type immortalized astrocytes (WT5) (Figure 5). It is reported that reactive astrocytes exhibit an increased production of CSPG as well as GFAP, vimentin, and nestin (McKeon et al., 1999). The CSPG that constitutes a part of the astroglial scar is known to inhibit the differentiation of oligodendrocyte precursor cells *in vitro* and to be an impediment to remyelination *in vivo* (Groves et al., 1993; Langley et al., 2009; Lau et al., 2012). Therefore, *ABCD1*-deficient astrocytes in cerebral ALD may be involved in the failure of remyelination as the result of producing CSPG.

In conclusion, we have established immortalized *Abcd1*-deficient astrocytes that display phenotypic characteristics that resemble those of reactive astrocytes. The results in this study indicate that they are autonomously primed for inflammation without the participation of microglia. ABCD1-deficiency induces the accumulation of lipid molecules containing VLCFA. VLCFA accumulation is suggested to cause mitochondrial dysfunction and in turn the production of reactive oxygen species (Di Cara et al., 2019), which may lead to a pro-inflammatory state in astrocytes. It is reported that the activation of transcription factors such as NF-κB by LPS could be the driving force for the inflammation in *Abcd1*-silenced mouse primary astrocytes (Singh et al., 2009). Although the precise mechanism underlying the entry into the pro-inflammatory state are unknown, certain protein kinases and inhibitory factors that are involved in the NF-κB signaling pathway might be dysregulated in *Abcd1*-deficient astrocytes. Identification of the mechanism underlying the pro-inflammatory state of astrocytes in ABCD1-deficiency will be required to understand the cellular and molecular pathology of X-ALD.

## Declarations

### Author contribution statement

Masashi Morita: Conceived and designed the experiments; Performed the experiments; Analyzed and interpreted the data; Wrote the paper.

Ai Toida, Yuki Horiuchi: Performed the experiments; Analyzed and interpreted the data.

Shiro Watanabe: Analyzed and interpreted the data; Contributed reagents, materials, analysis tools or data.

Masakiyo Sasahara: Contributed reagents, materials, analysis tools or data.

Kosuke Kawaguchi: Analyzed and interpreted the data.

Takanori So: Contributed reagents, materials, analysis tools or data; Wrote the paper.

Tsuneo Imanaka: Conceived and designed the experiments; Analyzed and interpreted the data; Wrote the paper.

### Funding statement

This work was supported in part by a Grant-in-Aid for Scientific Research from the 10.13039/501100001700Ministry of Education, Culture, Sports, Science and Technology (19K08271).

### Data availability statement

Data included in article/supplementary material/referenced in article.

### Declaration of interests statement

The authors declare no conflict of interest.

### Additional information

No additional information is available for this paper.

## Appendix A Supplementary data

Supplementary Fig Legend revised Supplementary Fig Legend revised

Supplementary Fig revised _spl_Morita et al_spl_ Supplementary Fig revised _spl_Morita et al_spl_
