# Supplementary material for: Generation of an immortalized astrocytic cell line from Abcd1-deficient H-2KbtsA58 mice to facilitate the study of the role of astrocytes in X-linked adrenoleukodystrophy
Source: Heliyon. 2021 Feb 11;7(2):e06228. doi: 10.1016/j.heliyon.2021.e06228 (PMC7892932; doi:10.1016/j.heliyon.2021.e06228)
Supplement: Supplementary Fig Legend revised [file mmc1.docx]

**Supplementary Fig. 1 Preparation of immortalized male mice for establishing stable cell lines**

Female *Abcd1*-deficient (homozygous) mice were mated with male H-2k^b^tsA58 transgenic mice to generate the *Abcd1*-deficient H-2k^b^tsA58 transgenic mouse. Mixed glial cells were prepared from 1- to 2-day old mice. The expression of the *Sry* and *H-2Kb* genes was determined by PCR. The PCR products were separated by agarose gel electrophoresis and detected under UV light. A mixed glial culture expressing both the *Sry* and *H-2Kb* genes, such as No. 4, 6, 8, 13 and 14, was used for cloning. The figure shows a representative result.

**Supplementary Fig. 2 Original full images of immunoblots**
