# Supplementary material for: Generation of an immortalized astrocytic cell line from Abcd1-deficient H-2KbtsA58 mice to facilitate the study of the role of astrocytes in X-linked adrenoleukodystrophy
Source: Heliyon. 2021 Feb 11;7(2):e06228. doi: 10.1016/j.heliyon.2021.e06228 (PMC7892932; doi:10.1016/j.heliyon.2021.e06228)
Supplement: Supplementary file 2 [file mmc2.docx]

## Slide 1

Sry

H-2Kb 

1    2    3    4    5    6    7    8    9  10  11  12  13  14

Suppl. Fig. 1 Morita et al

Mixed glial culture

## Slide 2

Abcd3

Abcd1

Abcd2

Actin

Suppl. Fig. 2 Morita et al

Primary

Immortalized

WT

KO

WT5

KO9

marker

Primary

Immortalized

WT

KO

WT5

KO9

marker

Primary

Immortalized

WT

KO

WT5

KO9

marker

Primary

Immortalized

WT

KO

WT5

KO9

marker

204

117

83

61

47

33

28

20

204

117

83

61

47

33

28

20

204

117

83

61

47

33

28

20

204

117

83

61

47

33

28

20

kDa

kDa

kDa

kDa

## Slide 3

Primary

Immortalized

WT

KO

WT5

KO9

marker

kDa

Ponceau-S 

204

117

83

61

47

33

28

20

Suppl. Fig. 2 Morita et al
